# Microvascular impairments detected by optical coherence tomography angiography in multiple sclerosis patients: A systematic review and meta-analysis

**DOI:** 10.3389/fnins.2022.1121899

**Published:** 2023-01-13

**Authors:** Jing Liu, Shuang Song, Xiaoya Gu, Hui Li, Xiaobing Yu

**Affiliations:** ^1^Department of Ophthalmology, Beijing Hospital, National Center of Gerontology, Institute of Geriatric Medicine, Chinese Academy of Medical Sciences, Beijing, China; ^2^Graduate School of Peking Union Medical College, Beijing, China

**Keywords:** multiple sclerosis, optic neuritis, retina, microvasculature, meta-analysis

## Abstract

**Purpose:**

A systematic review and meta-analysis was conducted to investigate changes in retinal and choroidal microvasculature in patients with multiple sclerosis (MS) using optical coherence tomography angiography (OCTA).

**Methods:**

PubMed and Google Scholar were searched for studies that compared retinal and choroidal microvasculature between MS and healthy controls (HC) with OCTA. MS patients were divided into 2 groups: MS with (MSON) or without optic neuritis (MSNON).

**Results:**

Totally, 13 studies including 996 MS eyes and 847 HC eyes were included. Compared with the HC, the vessel density of the whole superficial vascular complex (SVC) was reduced by 2.27% and 4.30% in the MSNON and MSON groups, respectively. The peripapillary vessel density was 2.28% lower and 4.96% lower in the MSNON and MSON groups, respectively, than in the HC. Furthermore, the MSON group had significant lower vessel density of the SVC (mean difference [MD] = −2.17%, *P* < 0.01) and lower peripapillary vessel density (MD = −2.02%, *P* = 0.02) than the MSNON group. No significant difference was found in the deep vascular complex or choriocapillaris densities among MSNON, MSON or HC groups (*P* > 0.05). Meta-regression analyses suggested that illness duration and the Expanded Disability Status Scale scores of MS patients were possible sources of heterogeneity (*P* < 0.05).

**Conclusion:**

The retinal SVC and peripapillary vessel density decreased significantly in MS eyes, especially in eyes with optic neuritis. Retinal microvasculature is a potential biomarker of disease progression in MS.

## 1. Introduction

Globally, about 2.5 million individuals are affected by multiple sclerosis (MS), one of the most prevalent causes of neurologic impairment in young to middle-aged adults (Oh et al., 2018; Thompson et al., 2018). In 25% of MS patients, optic neuritis (ON) is the initial symptom, and it develops in around 50% of individuals over the course of the disease (Di Maggio et al., 2014). However, the precise pathogenesis of MS is unclear and complicated. In addition to autoimmunity, vascular and metabolic factors have been gradually realized to play significant roles in the development of MS (Jiang et al., 2016; Zhang et al., 2018). MS has been viewed as a vascular disease due to the cerebral endothelial cells dysfunction and blood-brain barrier damage found in MS. Recent studies suggested that the blood-brain barrier disruption was an early change that can predict the conversion from ON to MS (Cramer et al., 2015). Phase-contrast magnetic resonance imaging (MRI) studies demonstrated MS patients had significantly decreased cervical arterial blood flow as compared to healthy controls (HC), consistent with the global cerebral hypoperfusion identified in MS brain (ElSankari et al., 2013; D'Haeseleer et al., 2015). Patients with MS have been reported to be more likely to develop ischemic heart disease, stroke, and peripheral vascular disease (Marrie et al., 2015).

Optical coherence tomography angiography (OCTA) is a novel imaging technique visualizing the retinal and choroidal microvasculature noninvasively (de Carlo et al., 2015). Without contrast agents or dye injection, OCTA images the microvasculature though detecting the motion contrast of blood cells (Kashani et al., 2017). As the retina shares similar embryonic origins, as well as anatomic and physiologic characteristics of the brain, it provides a potential window to detect cerebral pathologic changes in neurodegeneration diseases (Gupta et al., 2021). Optical coherence tomography (OCT) studies have reported the atrophy of the retinal nerve fiber layer (RNFL), ganglion cell layer and inner plexiform layer (GCIPL) in MS (Petzold et al., 2017). Therefore, OCTA, an advancement of OCT imaging, may provide useful information for monitoring microvasculature and blood perfusion alterations in MS (Kleerekooper et al., 2020). Since the introduction of OCTA in the year of 2014, many studies have investigated the retinal and choroidal microvascular alterations of MS patients with OCTA (Lanzillo et al., 2018; Feucht et al., 2019; Yilmaz et al., 2020; Aly et al., 2021).

Here, we present a meta-analysis to summarize the retinal and choroidal microvascular changes measured by OCTA in patients with MS. The microvasculature changes caused by clinically apparent ON associated with MS (MSON) are carefully distinguished from those in MS patients without optic neuritis (MSNON). We also further explored the roles of age, illness duration and severity in the retinal microvasculature in patients with MS.

## 2. Methods

### 2.1. Search strategy

PubMed and Google Scholar were searched for all studies that reported on the OCTA assessment of patients with MS using the following keywords: “optical coherence tomography angiography” OR “angio-OCT” OR “OCT angiography” OR “OCTA” combined with “multiple sclerosis” OR “MS”. The reference lists of all relevant articles were checked manually. No language or date restriction was applied during the search process. The final search of all the databases was conducted in 12 May 2022. This study followed the Meta-analysis Of Observational Studies in Epidemiology (MOOSE) guidelines (Stroup et al., 2000).

### 2.2. Inclusion and exclusion criteria

The inclusion criteria were studies including patients with MS and HC, using published consensus guidelines as diagnostic criteria of MS and MSON, and reporting OCTA outcomes of the study participants. Studies were excluded if they did not separate MSON from MSNON eyes, did not include HC, included duplicate study populations, lacked the data that could be extracted for analyses, or were review articles or animal studies.

### 2.3. Data extraction and assessment of study quality

Two reviewers (JL, SS) extracted the data and assessed the study quality independently, and resolved disagreements by consensus or consultation with a third reviewer (XY). If the requisite data were unavailable, the original authors of relevant studies were contacted. For each included study, the following information was extracted: author, title, publication year, country, study design, sample size, illness duration and the Expanded Disability Status Scale (EDSS) scores of patients with MS, mean age and sex of the participants, OCTA device used, and OCTA scanning patterns and outcomes. The outcomes included the vessel density of superficial (SVC) and deep vascular complex (DVC), peripapillary vessel density, and the vessel density of choriocapillaris. The vessel densities of the SVC, DVC, and choriocapillaris were scanned within a 3 × 3 or 6 × 6 mm square centered on the fovea, while the peripapillary vessel density centered on the optic disc. Due to the variety of image dividing methods in each study, all the vessel density data we extracted referred to the whole image. In the prospective longitudinal study, data were extracted at a single time point. The quality of observational studies was evaluated by the 22-item STROBE Statement checklist (von Elm et al., 2014).

### 2.4. Statistical analysis

STATA version 16.0 (StataCorp, College Station, TX) was used to analyze the data. We chose the random-effect model with the DerSimonian and Laird method to pool the estimates. For continuous variables, the mean difference (MD) and 95% confidence interval (CI) were calculated. Sensitivity analyses were performed by removing each study one by one. The *I*^2^ statistic was employed to evaluate the between-study heterogeneity. In order to identify probable causes of heterogeneity, meta-regression analyses were carried out. Publication bias was measured by funnel plots and Egger's regression test. *P* < 0.05 was considered statistically significant.

## 3. Results

### 3.1. Included studies and main characteristics

**Figure 1:**
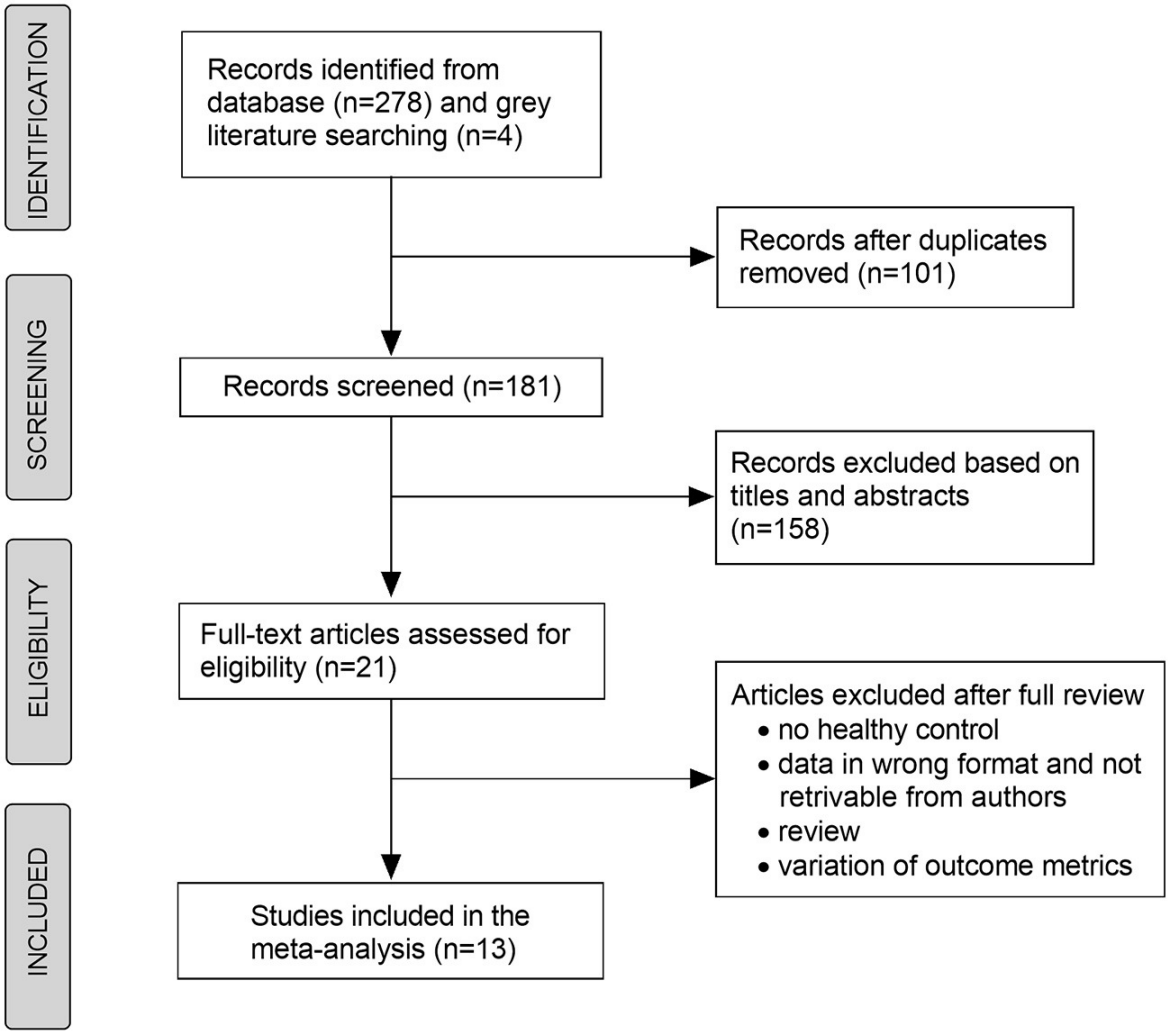
Flow chart depicting the selection of the studies included in the meta-analysis.

In total, 181 records were selected for title and abstract reading, from which 21 articles were eligible for full-text review (Figure 1). Rogaczewska et al. reported the results of the different outcome metrics in two papers based on the same HC group (Rogaczewska et al., 2021a,b). Therefore, we combined these results into a single study. Ultimately, this meta-analysis included 13 studies with 996 MS eyes and 847 HC eyes (Lanzillo et al., 2018; Feucht et al., 2019; Cennamo et al., 2020; Cordon et al., 2020; Farci et al., 2020; Murphy et al., 2020; Ulusoy et al., 2020; Yilmaz et al., 2020; Aly et al., 2021; Kallab et al., 2021; Khader et al., 2021; Montorio et al., 2021; Rogaczewska et al., 2021a,b). Murphy et al. (2020) reported the results stratified by image artifact grading, and we only included the data of minimal artifact in the quantitative analysis of meta-analysis. Among 13 included studies, four studies (Cordon et al., 2020; Farci et al., 2020; Kallab et al., 2021; Khader et al., 2021) analyzed monocular data, whereas nine studies (Lanzillo et al., 2018; Feucht et al., 2019; Cennamo et al., 2020; Murphy et al., 2020; Ulusoy et al., 2020; Yilmaz et al., 2020; Aly et al., 2021; Montorio et al., 2021; Rogaczewska et al., 2021a,b) analyzed biocular data. For patients with MS, the mean illness duration ranged from 4 to 11 years and the mean EDSS scores ranged from 1.0 to 3.5. The general characteristics of the included studies are presented in Table 1.

**Table 1:** Characteristics of the included studies.

**Study, year**	**Country**	**No. of eyes**		**Age (year)**		**Sex (Male%)**		**Duration of MS (year)**	**EDSS scores**	**OCTA device**	**STROBE scores**
		**Control group**	**MS group**	**Control group**	**MS group**	**Control group**	**MS group**				
Aly et al. (2021)	Germany	42	41	42.0	38.0	23.8	23.8	5.7	1.4	OptoVue	20
Cennamo et al. (2020)	Italy	30	20	28.2	29.7	33.3	30.0	4.0	2.3	OptoVue	19
Cordon et al. (2020)	Spain	149	92	41.8	41.7	13.4	13.0	5.0	2.0	Topcon	18
Farci et al. (2020)	Italy	29	91	52.7	41.5	65.5	13.2	NA	NA	OptoVue	18
Feucht et al. (2019)	Germany	100	83	32.0	30.0	68.0	69.0	4.1	1.0	OptoVue	20
Kallab et al. (2021)	Austria	18	32	41.0	43.0	22.2	25.0	9.0	NA	Heidelberg	19
Khader et al. (2021)	Egypt	10	20	30.0	30.9	NA	NA	4.3	NA	Zeiss	18
Lanzillo et al. (2018)	Italy	92	100	43.3	40.6	47.8	38.0	11.0	3.5	OptoVue	21
Montorio et al. (2021)	Italy	30	30	27.2	28.4	53.3	46.7	NA	1.8	OptoVue	20
Murphy et al. (2020)	USA	97	201	34.5	40.0	42.0	19.8	10.0	1.5	Heidelberg	20
Rogaczewska et al. (2021a,b)	Poland	40	77	37.9	35.2	15.0	20.0	8.0	NA	Optovue	17
Ulusoy et al. (2020)	Turkey	48	40	41.4	43.8	37.5	35.0	9.5	2.3	OptoVue	20
Yilmaz et al. (2020)	Turkey	122	94	38.6	38.3	19.7	17.0	8.2	1.5	Nidek	19

MS, Multiple sclerosis; EDSS, Expanded Disability Status Scale; OCTA, Optical coherence tomography angiography;NA, not available.

### 3.2. Outcome measures

All pooled estimates are summarized in Table 2.

**Table 2:** Alterations of retinal and choroidal microvascular density detected by OCTA in multiple sclerosis patients with or without optic neuritis.

**Outcome variables**	**No. of studies**	**No. of eyes**	**Weighted mean difference (95% CI)**	**P-value**	***I*^2^ test**	**Egger's test**
**SVC density (%)**						
MSNON vs. HC	11	1188	−2.27 (−3.12, −1.43)	< 0.01^**^	72.79%	0.19
MSON vs. HC	9	924	−4.30 (−5.27, −3.33)	< 0.01^**^	62.90%	0.85
MSON vs. MSNON	9	688	−2.17 (−3.11, −1.22)	< 0.01^**^	52.19%	0.61
**DVC density (%)**						
MSNON vs. HC	9	812	−0.79 (−2.05, 0.46)	0.21	78.11%	0.99
MSON vs. HC	7	652	−0.62 (−2.99, 1.74)	0.60	91.28%	0.54
MSON vs. MSNON	7	502	−0.21 (1.32, 0.89)	0.71	50.73%	0.71
**Peripapillary vessel density (%)**						
MSNON vs. HC	8	713	−2.82 (−4.57, −1.07)	< 0.01^**^	89.71%	0.02^*^
MSON vs. HC	6	534	−4.96 (−8.95, −0.97)	< 0.01^**^	96.63%	0.34
MSON vs. MSNON	7	444	−2.02 (−3.67, −0.36)	< 0.01^**^	70.61%	0.34
**Choriocapillaris density (%)**						
MSNON vs. HC	4	327	1.44 (−0.34, 3.21)	0.11	82.98%	< 0.01^**^
MSON vs. HC	2	215	5.59 (−3.79, 14.98)	0.24	95.95%	0.49
MSON vs. MSNON	2	174	0.62 (−0.21, 1.44)	0.14	< 0.01%	0.69

* *P* < 0.05,

** *P* < 0.01.

Eleven studies (Lanzillo et al., 2018; Feucht et al., 2019; Cennamo et al., 2020; Cordon et al., 2020; Farci et al., 2020; Murphy et al., 2020; Ulusoy et al., 2020; Yilmaz et al., 2020; Aly et al., 2021; Montorio et al., 2021; Rogaczewska et al., 2021b), which included 1,455 eyes, reported data on the vessel density of the SVC (Supplementary Figure 1). The SVC density was reduced by 2.27% (95% CI −3.12 to −1.43%, *P* < 0.01) and 4.30% (95% CI −5.27 to −3.33%, *P* < 0.01) in the MSNON and MSON groups, respectively, compared with that in the HC group. Furthermore, the MSON group had a significantly lower SVC density than the MSNON group by 2.17% (95% CI −3.11 to−1.22%, *P* < 0.01). By excluding studies in turn, sensitivity analyses showed the results remained consistent.

Nine studies (Feucht et al., 2019; Cennamo et al., 2020; Farci et al., 2020; Murphy et al., 2020; Ulusoy et al., 2020; Yilmaz et al., 2020; Aly et al., 2021; Montorio et al., 2021; Rogaczewska et al., 2021b), which included 1,038 eyes, reported data on the vessel density of the DVC (Supplementary Figure 2). No significant difference in the DVC density was found between the MSNON and HC groups (MD = −0.79%, *P* = 0.21). Sensitivity analysis suggested that the DVC density was significantly lower in the MSNON group than in the HC group by 1.28% (95% CI −2.25 to −0.31%, *P* = 0.01) after omitting the study conducted by Rogaczewska et al. (2021b). The differences in the DVC density between the MSON and HC groups (95% CI −2.99 to 1.74%, *P* = 0.60) and between the MSNON and MSON groups (95% CI −1.32 to 0.89%, *P* = 0.71) were not statistically significant.

Nine studies (Cennamo et al., 2020; Cordon et al., 2020; Farci et al., 2020; Ulusoy et al., 2020; Yilmaz et al., 2020; Kallab et al., 2021; Khader et al., 2021; Montorio et al., 2021; Rogaczewska et al., 2021a), which included 941 eyes, reported data on the peripapillary vessel density (Supplementary Figure 3). The peripapillary density was 2.82% (95% CI −4.57 to −1.07%, *P* < 0.01) and 4.96% (95% CI −8.95 to −0.97%, *P* = 0.01) lower in the MSNON and MSON groups, respectively, than in the HC group. Furthermore, the MSON group had a significant lower peripapillary density than the MSNON group (MD = −2.02%, 95% CI −3.67 to −0.36%, *P* = 0.02). Sensitivity analysis indicated that no single study had a substantial impact on the pooled results.

Four studies (Feucht et al., 2019; Cennamo et al., 2020; Farci et al., 2020; Montorio et al., 2021), which included 413 eyes, reported data on the choriocapillaris vessel density (Supplementary Figure 4). There was no significant difference in the choriocapillaris density between the MSNON and HC groups (MD = 1.44%, 95% CI −0.34 to 3.21%, P = 0.11), between the MSON and HC groups (MD = 5.59%, 95% CI −3.79 to 14.98%, *P* = 0.24), or between the MSON and MSNON groups (MD = 0.62%, 95% CI −0.21 to 1.44%, *P* = 0.14). Sensitivity analysis suggested that the pooled results were stable.

### 3.3. Assessment of heterogeneity and publication bias

**Figure 2:**
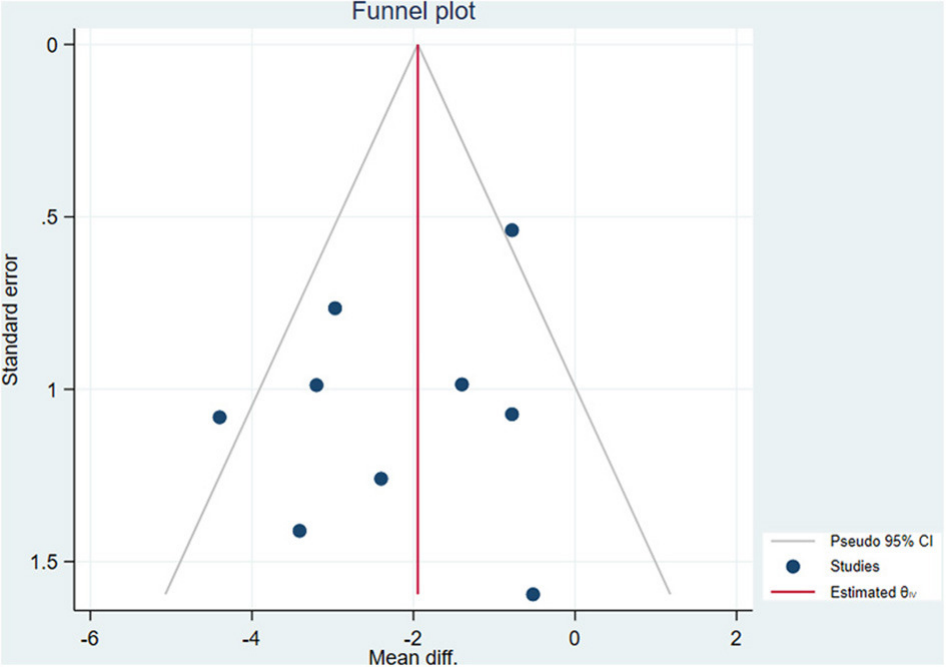
Funnel plot of the meta-analysis.

We then carried out meta-regression analyses to explore the roles of age, disease duration, EDSS scores, and the instrument type in the microvascular alterations of patients with MS (Supplementary Table 1). Age (*P* = 0.003) and the instrument type (*P* = 0.001) were sources of heterogeneity in the analyses of peripapillary vessel density and vessel density of the DVC, respectively. Disease duration and EDSS scores influenced the change in the SVC density significantly (*P* < 0.05). Regarding the outcomes of the vessel density of the choriocapillaris, meta-regression analyses were not performed because of the limited quantity of included studies. The shape of the funnel plot did not show any evidence of obvious asymmetry (Figure 2). Egger's test showed that no significant publication bias was detected in most of the comparisons (*P* > 0.05), except for the results of peripapillary vessel density (*P* = 0.02) and the vessel density of the choriocapillaris (*P* = 0.001) in those with MSNON eyes (Table 2).

## 4. Discussion

This is the first meta-analysis that, to our knowledge, integrates all the data needed to summarize the retinal and choroidal microvascular changes that were measured by OCTA in MS patients who have had ON or not. Our study demonstrated that the SVC and peripapillary vessel densities significantly decreased in both MSNON and MSON group than in the HC group. Furthermore, MSON eyes had lower SVC and peripapillary vessel densities than MSNON eyes.

OCTA provides high-resolution, non-dye visualization of retinal, choroidal, and peripapillary microvasculature, becoming a prominent noninvasive tool to aid in diagnosis and monitoring disease progression. The vessel density reported in the OCTA studies refers to the percentage of the blood flow area, also called perfusion density. Our meta-analysis showed that the macular SVC and peripapillary vessel densities, compared with those in the healthy controls, were reduced in patients with MS, whether they had ON or not. The findings within the retina are consistent with the results of imaging studies that show reduced cervical arterial blood flow and diffuse cerebral hypoperfusion in MS patients (Di Maggio et al., 2014; Cramer et al., 2015). This is hardly surprising given that the retina can be considered a developmental and structural extension of the CNS as it shares similar embryonic origins, as well as anatomic and physiologic characteristics of the brain (London et al., 2013). Furthermore, the results showed that the SVC and peripapillary vessel densities were significantly lower in MSON eyes than in MSNON eyes, suggesting that the macular and peripapillary microvascular alterations may be greater due to MS-associated ON (Murphy et al., 2020).

It is well established that the inner layers of the retina, such as the RNFL and GCIPL, are thinner in patients with MS than in age-matched individuals, and those changes are more pronounced in MS patients with a history of ON (Petzold et al., 2017). The SVC supplied the inner retinal layers, and the reduced SVC vessel density was consistent with atrophy of the RNFL and GCIPL (Yilmaz et al., 2020; Khader et al., 2021). Therefore, it is possible that the reduced SVC vessel density was secondary to RNFL atrophy and ganglion cell loss (Murphy et al., 2020). However, an alternate hypothesis is that microvascular alteration is a possible primary contributor to the pathogenesis of MS. This hypothesis is supported by the findings of brain imaging studies, which suggests that perfusion abnormalities can occur independently of gray matter volume atrophy (Zhang et al., 2018). Future research is needed to determine the specific involvement of microvascular abnormalities in the development of MS. The retina can, undoubtedly, be a useful “window” for addressing these questions.

Interestingly, the results of our study showed that unlike the SVC density, the DVC density did not change significantly in the MSNON or MSON groups. A possible explanation for the discordance of the changes in SVC and DVC densities was associated with the adaptation of retinal vessels to metabolic demand. Anatomically, the SVC supplies the RNFL and GCIPL layers, and the DVC supplies the inner nuclear layer (INL) and outer plexiform layer (OPL) (Petzold et al., 2017). As mentioned above, the RNFL and GCIPL layers atrophied in MS patients, while the thickness of the INL or OPL layer showed no significant change. Therefore, SVC density tended to be lower than DVC density because of atrophy of the RMFL and GCIPL layers and the consequent reduction in metabolic demand.

Heterogeneity, especially clinical heterogeneity, is an issue that should be considered in the meta-analysis (Engels et al., 2000). By meta-regression analyses, we found that illness duration and EDSS scores were two significant sources of heterogeneity, which indicated that changes in the retinal vessel density were influenced by the illness duration and global impairment levels (as assessed by EDSS score) of MS. These outcomes agreed with those of earlier clinical investigations, which reported that the SVC density negatively correlated with EDSS scores and illness duration, suggesting the roles of retinal microvessels in the process of neurodegeneration in MS (Murphy et al., 2020).

Inevitably, there are limitations to this study. First, the sensitivity analyses suggested that after omitting the study conducted by Rogaczewska et al. (2021b), the results of the DVC density could change. Therefore, this conclusion needs to be interpreted with some caution. Second, even though the meta-regression and sensitivity analyses were carefully conducted, substantial heterogeneities still existed in several outcomes. This is probably due to the cross-sectional nature of the data analyzed in the current meta-analysis. Therefore, the random-effect model was employed in the analyses to avoid overestimation. Moreover, the cross-sectional nature precludes causal conclusions. Prospective longitudinal studies are needed to explore the changes in retinal microvasculature throughout the disease process.

To conclude, this meta-analysis showed that the macular SVC and peripapillary vessel density significantly decreased in MS, and this change was more pronounced in MSON. In addition, changes in retinal microvessels were associated with the illness duration and disability levels of MS. These results confirmed retinal microvascular changes in MS. Large prospective studies are needed to investigate whether retinal microvascular structure, as assessed by OCTA, can be used as a noninvasive biomarker of disease diagnosis and progression in MS.

## Data availability statement

The original contributions presented in the study are included in the article/Supplementary material, further inquiries can be directed to the corresponding author.

## Author contributions

Study concept and design: JL, XY, and XG. Search strategy and first drafting of the manuscript: SS and JL. Acquisition, analysis or interpretation of data, and statistical analysis: JL, SS, and HL. Tables and figures: HL and JL. Critical revision for important intellectual content and final approval of the manuscript: XY and XG. All authors contributed to the article and approved the submitted version.
