# Supplementary material for: Microvascular impairments detected by optical coherence tomography angiography in multiple sclerosis patients: A systematic review and meta-analysis
Source: Front Neurosci. 2023 Jan 13;16:1121899. doi: 10.3389/fnins.2022.1121899 (PMC9880267; doi:10.3389/fnins.2022.1121899)
Supplement: Supplementary file 1 [file Table_1.DOCX]

Supplementary Material

# Supplementary Figures

**Supplementary Figure 1.** Forest plots of the vessel density of superficial vascular complex in patients (A) without and (B) with a history of optic neuritis, and (C) comparison of MSON and MSNON eyes.

**Supplementary Figure 2.** Forest plots of the vessel density of deep vascular complex in patients (A) without and (B) with a history of optic neuritis, and (C) comparison of MSON and MSNON eyes.

**Supplementary Figure 3.** Forest plots of the peripapillary vessel density in patients (A) without and (B) with a history of optic neuritis, and (C) comparison of MSON and MSNON eyes.

**Supplementary Figure 4**. Forest plots of the vessel density of choriocapillaris in patients (A) without and (B) with a history of optic neuritis, and (C) comparison of MSON and MSNON eyes.

# 2 Supplementary Tables

# **Supplementary Table 1**. Meta-regression analyses of effects of age, duration, EDSS scores and instrument.

| Outcome variables | Groups | Variable | Coefficient | Standard error | 95% CI | P |
| --- | --- | --- | --- | --- | --- | --- |
| the vessel density of SVC | MSNON vs. HC | Age | 0.20 | 0.12 | -0.04 to 0.44 | 0.109 |
|  |  | Duration | -0.01 | 0.02 | -0.04 to 0.03 | 0.798 |
|  |  | EDSS | -1.64 | 0.72 | -3.04 to -0.23 | 0.022* |
|  |  | Instrument | -0.04 | 0.32 | -0.66 to 0.58 | 0.898 |
|  | MSON vs. HC | Age | 0.24 | 0.16 | -0.08 to 0.55 | 0.139 |
|  |  | Duration | -0.06 | 0.02 | -0.10 to -0.01 | 0.012* |
|  |  | EDSS | 0.75 | 0.90 | -1.02 to 2.52 | 0.407 |
|  |  | Instrument | -0.01 | 0.32 | -0.62 to 0.62 | 0.994 |
|  | MSON vs. MSNON | Age | 0.07 | 0.15 | -0.21to 0.36 | 0.618 |
|  |  | Duration | -0.04 | 0.01 | -0.07 to -0.01 | 0.004** |
|  |  | EDSS | 1.73 | 0.77 | 0.22 to 3.25 | 0.025* |
|  |  | Instrument | -0.06 | 0.29 | -0.63 to 0.51 | 0.833 |
| the vessel density of DVC | MSNON vs. HC | Age | 0.15 | 0.24 | -0.32to 0.62 | 0.529 |
|  |  | Duration | 0.01 | 0.03 | -0.06 to 0.07 | 0.977 |
|  |  | EDSS | -1.52 | 1.21 | -3.90 to 0.85 | 0.209 |
|  |  | Instrument | -0.85 | 0.26 | -1.36 to -0.34 | 0.001** |
|  | MSON vs. HC | Age | 1.02 | 0.99 | -0.91 to 2.96 | 0.300 |
|  |  | Duration | -0.04 | 0.11 | -0.25 to 0.17 | 0.681 |
|  |  | EDSS | 1.01 | 3.10 | -5.06 to 7.08 | 0.745 |
|  |  | Instrument | -1.53 | 0.95 | -3.41 to 0.33 | 0.107 |
|  | MSON vs. MSNON | Age | 0.90 | 0.94 | -0.96 to 2.73 | 0.337 |
|  |  | Duration | -0.05 | 0.10 | -0.24 to 0.15 | 0.640 |
|  |  | EDSS | 1.24 | 2.15 | -2.97 to 5.45 | 0.564 |
|  |  | Instrument | -0.69 | 0.91 | -2.48 to 1.11 | 0.452 |
| peripapillary vessel density | MSNON vs. HC | Age | 0.32 | 0.11 | 0.11 to 0.53 | 0.003** |
|  |  | Duration | 0.06 | 0.04 | -0.02 to 0.13 | 0.161 |
|  |  | EDSS | -2.70 | 4.67 | -11.86 to 6.46 | 0.564 |
|  |  | Instrument | 0.15 | 0.65 | -1.13 to 1.43 | 0.818 |
|  | MSON vs. HC | Age | 0.24 | 0.44 | -0.63 to 1.11 | 0.593 |
|  |  | Duration | 0.00 | 0.08 | -0.15 to 0.14 | 0.949 |
|  |  | EDSS | -1.08 | 1.44 | -3.91 to 1.75 | 0.455 |
|  |  | Instrument | -0.35 | 1.67 | -3.61 to 2.92 | 0.832 |
|  | MSON vs. MSNON | Age | -0.01 | 0.28 | -0.55 to 0.53 | 0.976 |
|  |  | Duration | 0.01 | 0.05 | -0.08 to 0.11 | 0.794 |
|  |  | EDSS | -2.93 | 1.84 | -6.55 to 0.68 | 0.112 |
|  |  | Instrument | -0.05 | 0.59 | -1.21 to 1.11 | 0.936 |

*P<0.05, **P<0.01
