# Supplementary figures and images for: Microvascular impairments detected by optical coherence tomography angiography in multiple sclerosis patients: A systematic review and meta-analysis
Source: Front Neurosci. 2023 Jan 13;16:1121899. doi: 10.3389/fnins.2022.1121899 (PMC9880267; doi:10.3389/fnins.2022.1121899)

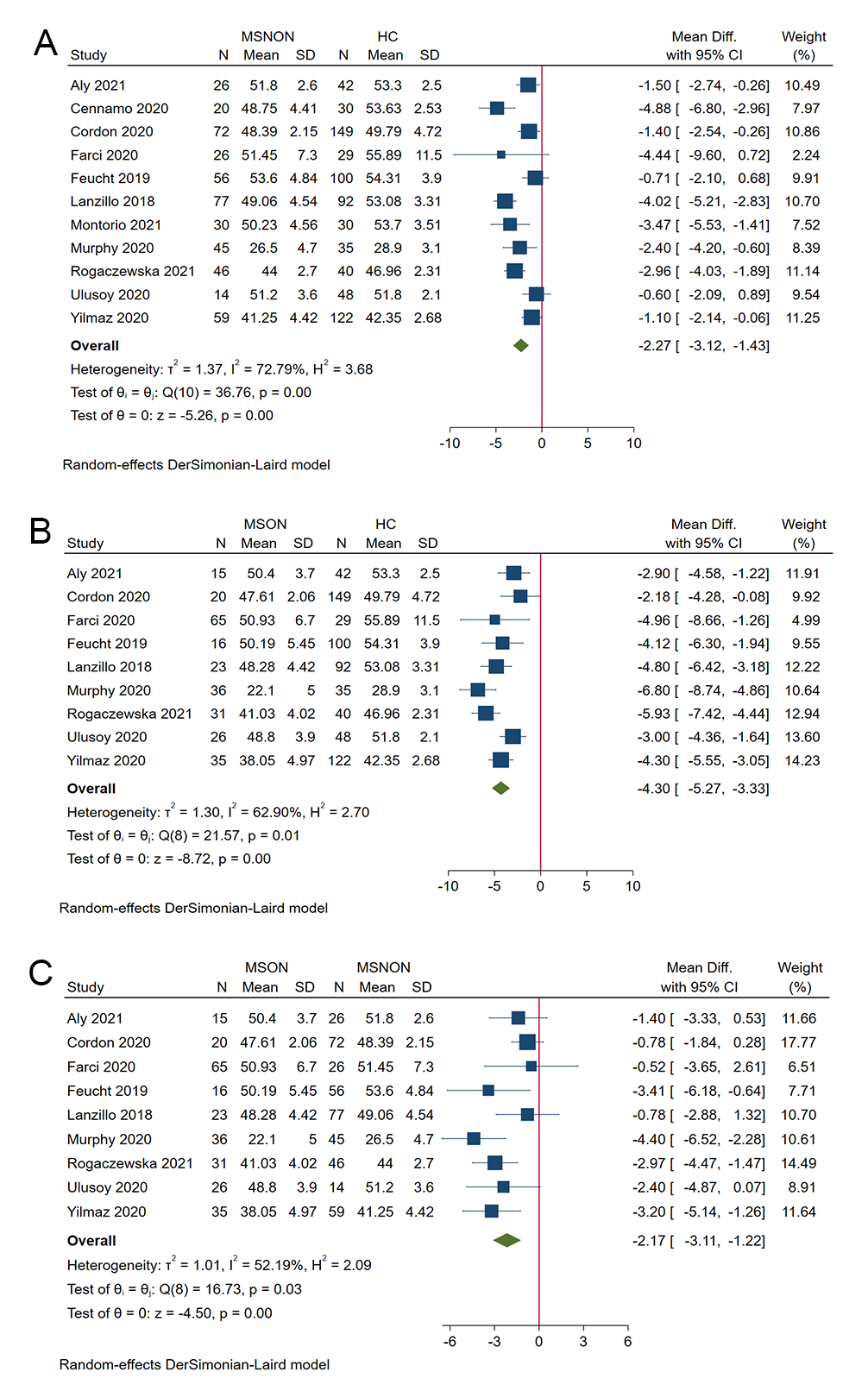

Supplement: Supplementary file 2 [file Image_1.TIF]

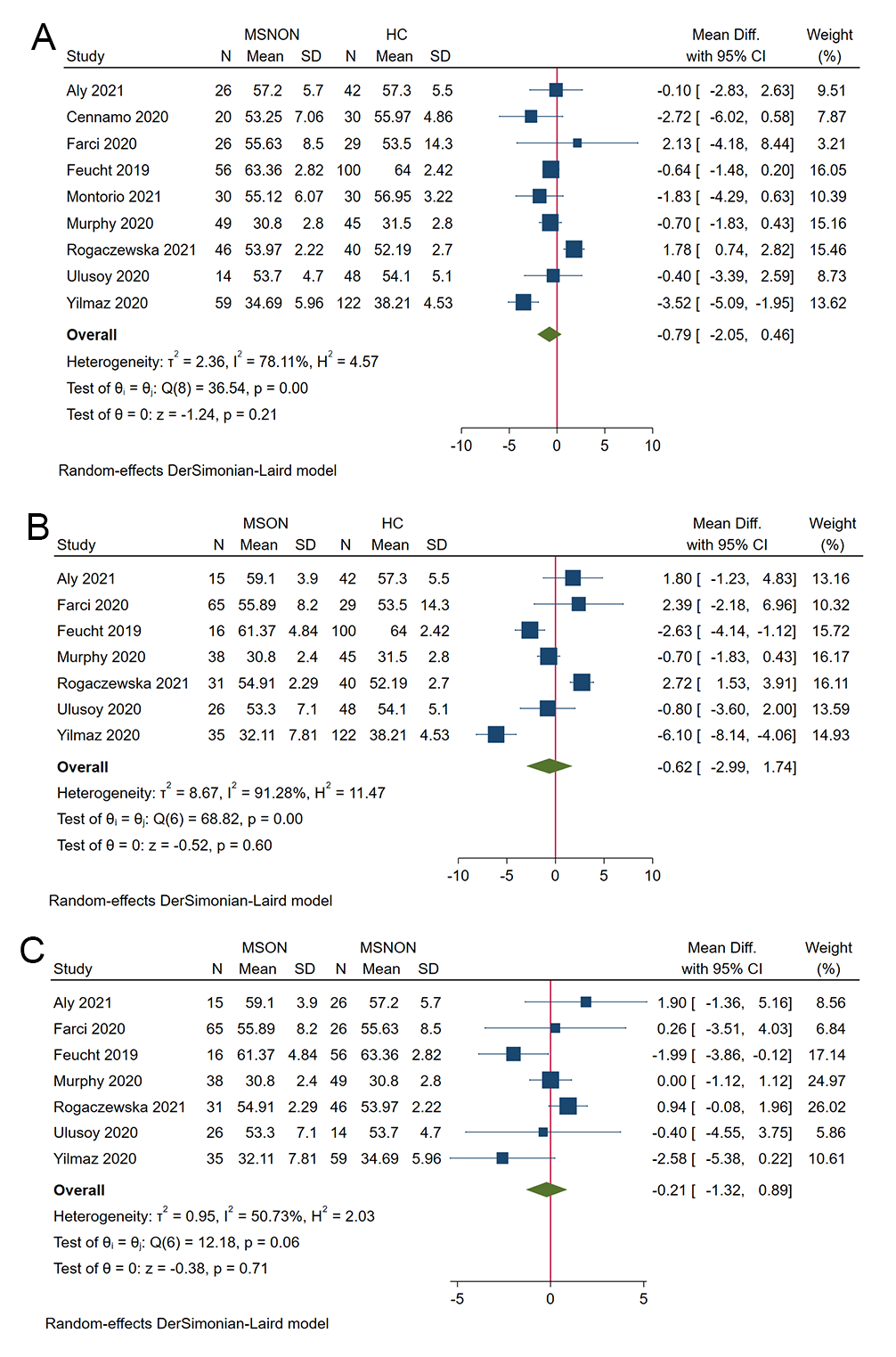

Supplement: Supplementary file 3 [file Image_2.TIF]

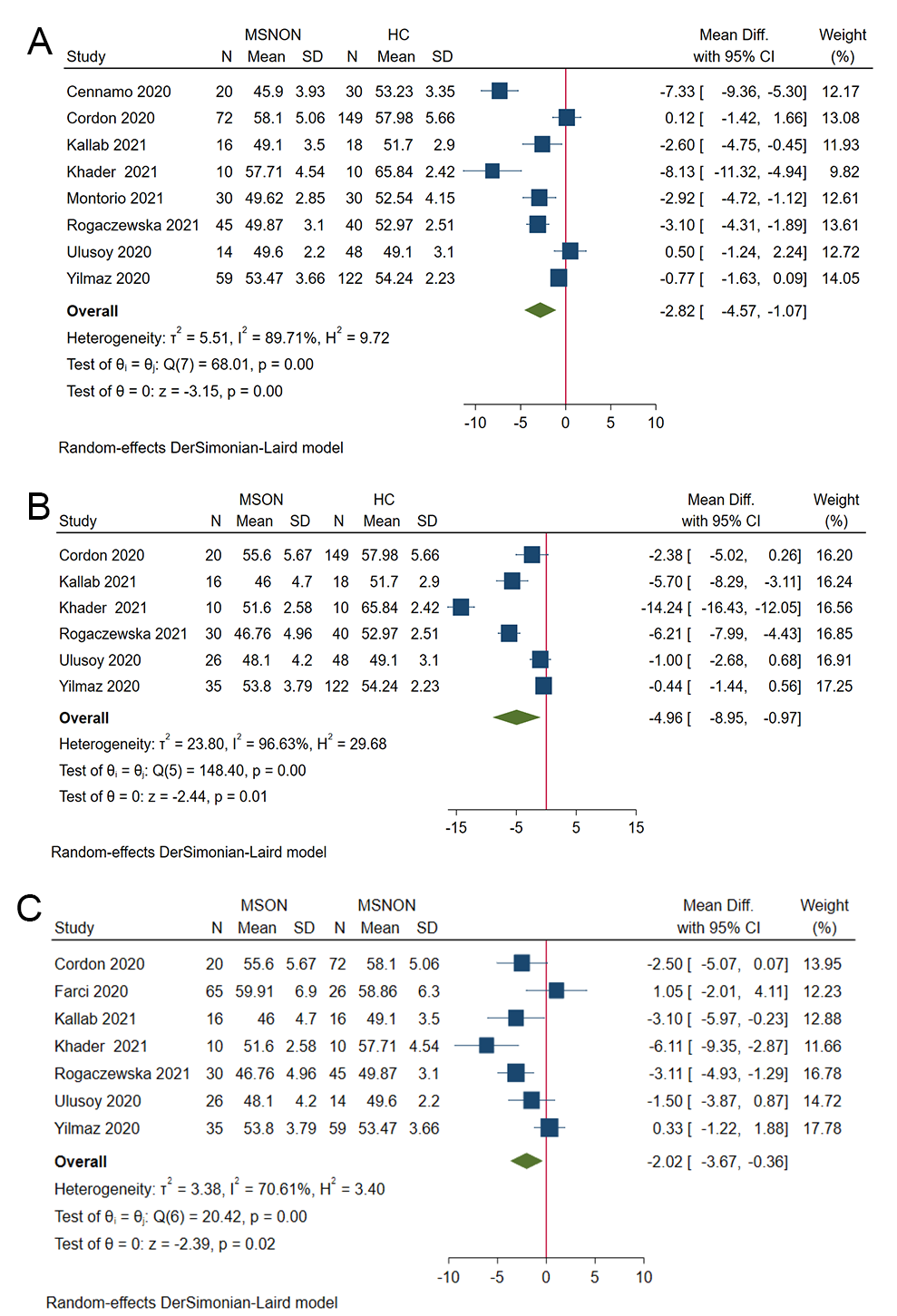

Supplement: Supplementary file 4 [file Image_3.TIF]

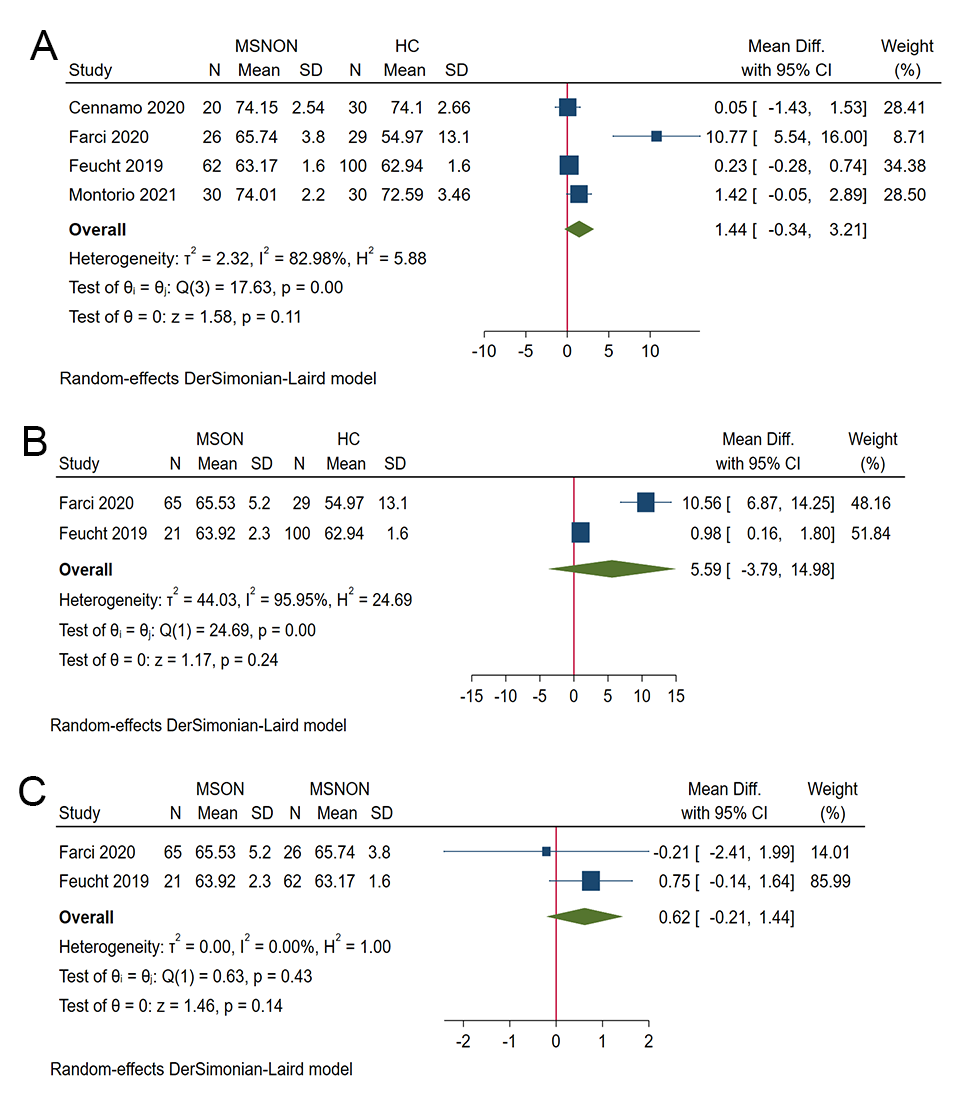

Supplement: Supplementary file 5 [file Image_4.TIF]
